# Recovery of Yam Soluble Protein from Yam Starch Processing Wastewater

**DOI:** 10.1038/s41598-020-62372-6

**Published:** 2020-03-25

**Authors:** Heng-Yue Xue, Yue Zhao, Zi-Heng Liu, Xiao-Wen Wang, Jun-Wei Zhang, Xue Peng, Masaru Tanokura, You-Lin Xue

**Affiliations:** 1 College of Light Industry, Liaoning University, Shenyang, 110036 P. R. China; 2 Department of Applied Biological Chemistry, Graduate School of Agricultural and Life Sciences, The University of Tokyo, Tokyo, 113-8657 Japan; 3 Dalian Institute for Drug Control, Dalian, 116021 P.R. China

**Keywords:** Proteins, Liquid chromatography

## Abstract

Over the past two decades, many studies have shown that the yam storage protein dioscorin, which is abundant in the wastewater of starch processing, exhibits many biological activities both *in vitro* and *in vivo*. In the present study, the acid-precipitation method was optimized using Box-Behnken design (BBD) combined with response surface methodology (RSM) for the recovery of yam soluble protein (YSP) from wastewater. The experimental yield of YSP reached 57.7%. According to relative quantitative proteomics (LC-MS/MS), the crude YSP was mainly composed of 15 dioscorin isoforms, which was further verified by anion-exchange and size-exclusion chromatography. YSP was found to be rich in glutamic acid and aspartic acid, and the eight essential acids made up approximately 33.7% of the YSP. Moreover, the YSP demonstrated antioxidant activity, including scavenging DPPH, hydroxyl and superoxide anion radicals, and the possible structure-activity relationships were discussed. These results indicated that YSP produced by acid precipitation may be used as a protein source with antioxidant properties.

## Introduction

The tubers of yams (*Dioscorea* spp.) are an important staple food in West Africa and Southeast Asia^1^. Yams have also been traditionally used as an herbal medicine and health food in Asia^2^. Yam tubers are composed of 75–84% starch, 6–8% crude protein and 1.2–1.8% crude fiber on a dry-weight basis^3^. Due to its desirable processing properties, yam starch is industrially produced with the generation of a large volume of liquid waste, which contains many high value byproducts, especially protein^4^.

Dioscorin is the major tuber storage protein of yams, accounting for 80–85% of the total tuber soluble protein. Over the past two decades, many studies have shown that dioscorin exhibits biological activity both *in vitro* and *in vivo*, including enzymatic, antioxidant, antihypertensive, immunomodulatory, lectin and airway epithelial cell-protecting activities^5,6^. However, the extraction and purification of the proteins from yams is time-consuming and environmentally unfriendly. The alkali extraction method (pH 8.3–8.5) was first used to extract dioscorin from lyophilized yam powder, and the protein was further purified by DE52 anion-exchange chromatography^7^. The alkali extraction method is still used today for the extraction of dioscorin from fresh yams. Conlan *et al*. purified obtained dioscorin by Sephadex G-75 chromatography^8^. In several studies, ammonium sulfate precipitation (saturation levels between 45–75%) and dialysis have been applied to reduce impurities before the chromatographic process (DE52, Sephadex G-75, Sephadex A-25 or Resource Q)^9–11^, which is time-consuming and produces lower yields. Furthermore, different bubble/foam fractionation systems have been designed for the recovery of mucilage, which contains approximately 50% soluble carbohydrates and 40% proteins, from fresh yams or starch-processing wastewater^4,12,13^. The systems are efficient for the recovery of yam mucilage that has a lower protein content.

Oxidation is a process crucial for the production of energy to fuel biological processes in living organisms. Excess oxidation produces free radicals, which can damage cells, thereby causing diseases, such as heart disease, inflammation, atherosclerosis and carcinogenesis^14^. Synthetic antioxidants have been used in food systems to retard the formation of free radicals, but recent studies have indicated that potential health hazards, such as liver damage and carcinogenesis, may be induced^15^. Thus, it is necessary to exploit and develop natural low toxicity antioxidants to protect the human body from radicals. It has been reported that dioscorin from *Dioscorea batatas* Decne showed scavenging activity against DPPH and hydroxyl radicals in a concentration-dependent manner^16^. Additionally, dioscorin from *Dioscorea alata* L. cv. Tainong 1 exhibited different scavenging activities against DPPH and hydroxyl radicals^17^. Furthermore, native and recombinant dioscorins form *Dioscorea japonica* Thunb. and *Dioscorea pseudojaponica* var. Keelung have shown DPPH radical scavenging activities^18^, which suggests that the variety and extraction method may affect the radical scavenging activity of the protein.

In the present study, yam soluble protein (YSP; from *Dioscorea opposita* Thunb.) was recovered from the wastewater of starch processing using the acid precipitation method. This method has been applied to the industrial extraction of large quantities of proteins, and it is different from the existing dioscorin extraction methods^6,19^. The extraction of YSP was optimized by response surface methodology (RSM) with Box-Behnken design (BBD), and the proximate composition, protein composition, anion-exchange chromatography, size exclusion chromatography, SDS-PAGE and the amino acid composition of YSP were investigated. Furthermore, the *in vitro* antioxidant activities, including DPPH radical-, hydroxyl radical- and superoxide radical-scavenging activities, were also evaluated.

## Results and Discussion

### Effect of extraction pH on the yield of YSP

**Figure 1:**
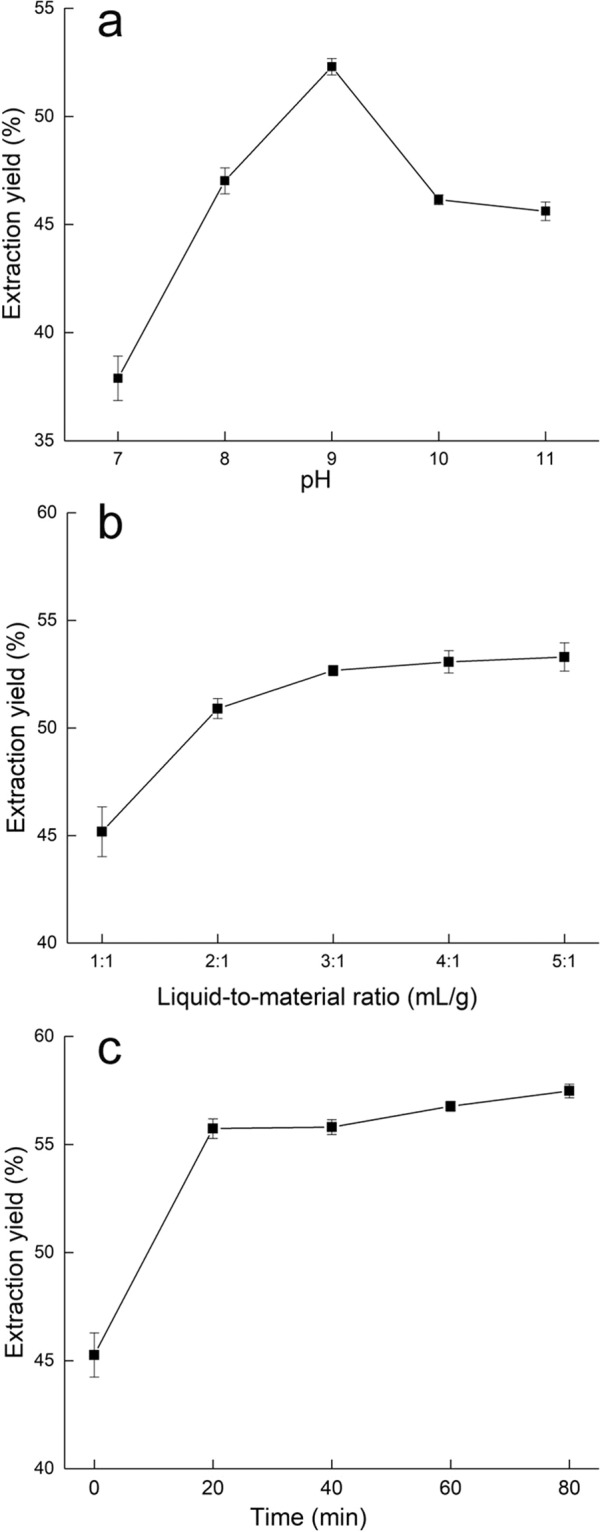
Effects of extraction pH (**a**), liquid-to-material ratio (**b**), and extraction time (**c**) on YSP yield. The error bars represent the standard deviations from three independent samples.

As shown in Fig. 1a, the effect of extraction pH (7, 8, 9, 10 and 11) on the yield of YSP was investigated when the extraction time and liquid-to-material ratio were fixed at 20 min and 3:1 mL/g, respectively. The yield increased to its maximum of 49.7% at pH 9 and then decreased. Protein molecules acquire more negative charges at alkaline pH values, which increases their intermolecular repulsion, thereby increasing protein solubility^20^. When the pH > 9, the protein extraction rate decreased, which may have been due to excessive protein hydrolysis.

### Effect of the ratio of liquid to material on the yield of YSP

As shown in Fig. 1b, the effect of the liquid-to-material ratio (1:1, 2:1, 3:1, 4:1 and 5:1 mL/g) on the yield of YSP was studied when the extraction pH and time were 9.0 and 20 min, respectively. The yield increased to its greatest amount of 52.7% at 3:1 mL/g and then gradually increased with increasing ratio, which may have been due to enhancement in the dilution effect, where the viscosity and protein concentration of the extracts decreased. Increased protein dissolution results in increased extraction yield^21^. When the liquid-to-material ratio was greater than 3:1 mL/g, the rate of diffusion decreased, resulting in a slower yield increase. Therefore, a 3:1 mL/g liquid-to-material ratio was used for the following experiments.

### Effect of extraction time on the yield of YSP

As shown in Fig. 1c, the effect of extraction time (0, 20, 40, 60 and 80 min) on the extraction yield of YSP was studied when the extraction pH and liquid-to-material ratio were fixed at 9.0 and 3:1 mL/g, respectively. The extraction yield reached 55.7% after 20 min and then gradually increased as the extraction continued. The results suggested that 40 min of extraction is sufficient to obtain the greatest extraction yield of YSP.

### Response surface analysis

The BBD experimental design and the results of 17 runs are presented in Supplementary Table 1. By analyzing the experimental data using multiple regression analysis, a predicted response Y for the yield of YSP was expressed by the following second-order polynomial equation:

$$\begin{array}{rcl}{R}_{1} & = & 52.55-5.83A+1.77B-0.97C-0.78AB-0.41AC\\  &  & +0.56BC-1.48{A}^{2}-3.82{B}^{2}+0.16{C}^{2}\end{array}$$

where R_1_ is the yield of YSP (%) and A, B and C are the coded factors of the tested variables: extraction pH, liquid-to-material ratio and time, respectively.

As shown in Table 1, the analysis of variance (ANOVA) of the response surface quadratic model indicated that the regression model obtained was highly significant (P < 0.0001) with an F value of 263.99. The lack of fit F value of 0.72 implied that the value was not significant compared to the pure error. The results showed that all investigated parameters had a notable effect on the extraction yield of YSP and could be ranked in decreasing order of importance in their effects as follows: extraction pH > liquid-to-material ratio > extraction time. The coefficient of determination (R^2^) obtained was 0.9971, suggesting that the obtained model adequately represented the real relationship among the selected parameters. These results demonstrated that the yield of YSP was affected by three independent factors (A, B and C), two interaction terms (AB and BC), and two quadratic terms (A^2^ and B^2^).

**Table 1:** Analysis of variance for the fitted regression model equation.

Source	Squares	df	Square	F-value	P-value; Prob > F	significant
Model	382.09	9	42.45	263.99	<0.0001	**
A	271.68	1	271.68	1689.37	<0.0001	**
B	25.03	1	25.03	155.63	<0.0001	**
C	7.54	1	7.54	46.88	0.0002	**
AB	2.43	1	2.43	15.13	0.0060	**
AC	0.66	1	0.66	4.09	0.0828	
BC	1.27	1	1.27	7.87	0.0263	*
A²	9.21	1	9.21	57.29	0.0001	**
B²	61.50	1	61.50	382.40	<0.0001	**
C²	0.11	1	0.11	0.71	0.4283	
Residual	1.13	7	0.16			
Lack of Fit	0.39	3	0.13	0.72	0.5904	not significant
Pure Error	0.73	4	0.18			
Cor Total	383.22	16				

Note: **Indicates significant differences (P < 0.01), *Indicates significant differences (P < 0.05).

The response surface and contour plots of the mutual interactions of the independent variables are shown in Supplementary Fig. 1. First, the effects of extraction pH and liquid-to-material ratio and their interaction on protein yield are presented in Supplementary Fig. 1a,b. The protein yield initially increased with increasing liquid-to-material ratio but decreased when the extraction pH and liquid-to-material ratio were further increased beyond a certain point. As shown in Supplementary Fig. 1a, a fractional elliptic contour was observed, suggesting the existence of significant and positive synergistic effects of extraction pH and liquid-to-material ratio on protein yield, which was in accordance with the results obtained from Supplementary Table 1. Supplementary Fig. 1c,d present the effects of the liquid-to-material ratio and extraction time and their interaction on the protein yield. The protein yield first increased with increasing liquid-to-material ratio but then decreased when the liquid-to-material ratio and extracting time further increased beyond a certain point.

The optimal extraction conditions for YSP were obtained when using the following conditions: liquid-to-material ratio of 3:1 mL/g, pH 8.5, and extraction for 20 min. Under the optimum conditions, the experimental YSP extraction yield reached 57.74%, which was similar to the predicted value (57.88%). The YSP extraction regression model was highly significant, indicating that it can be used to predict protein extraction yield.

### Proximate composition of YSP

The proximate composition of YSP was determined as follows: 65.3 ± 0.3% protein, 2.3 ± 0.1% fat, 1.6 ± 0.2% fiber, 3.3 ± 0.5% ash and 18.5 ± 1.1% total sugar (dry basis). The relatively high protein content of YSP (65.3%) justified its use as a candidate healthcare product, and the 18.5% total sugar content suggested that YSP is a glycoprotein^11^.

### Protein composition of YSP

**Figure 2:**
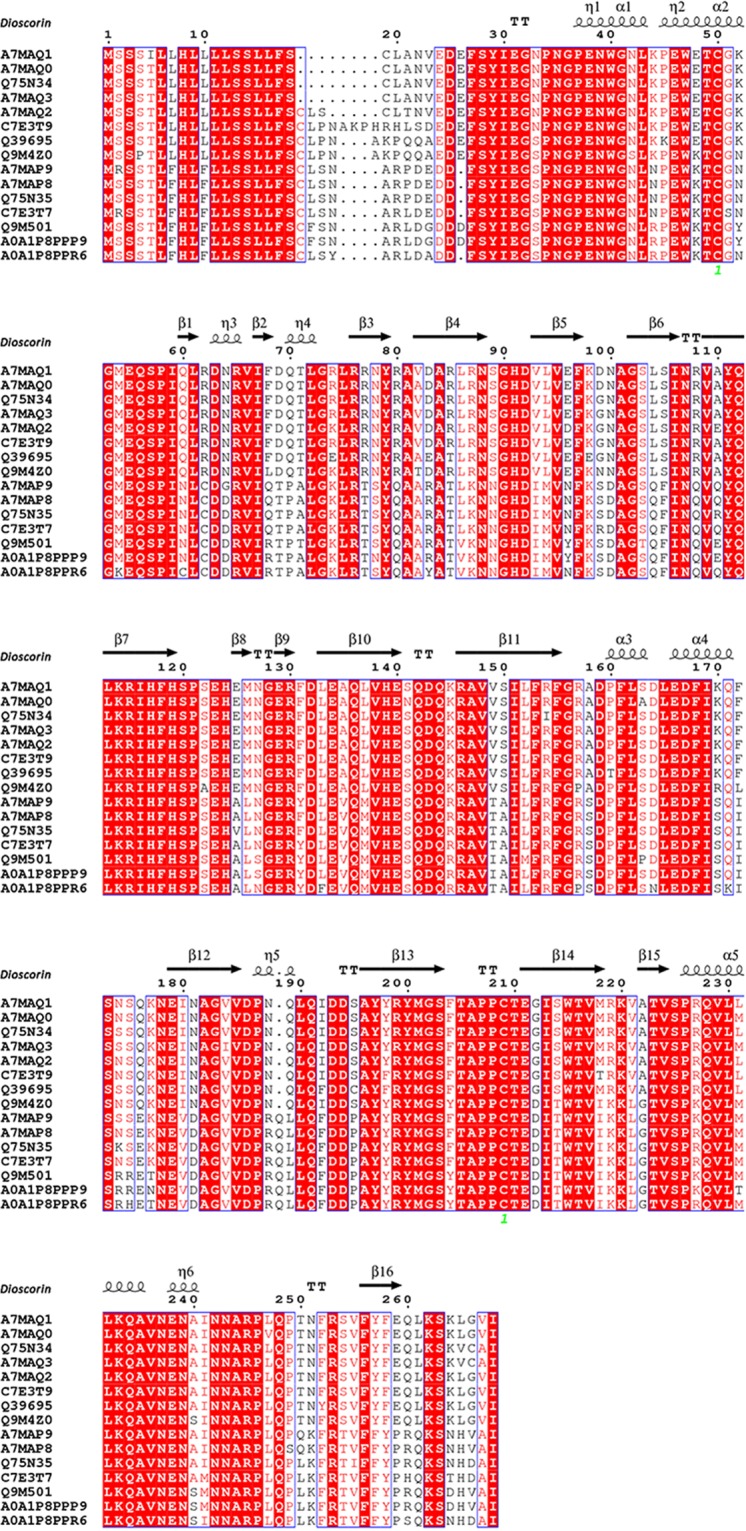
Sequence alignment of dioscorin isoforms from YSP using ClustalW2 (http://www.ebi.ac.uk/Tools/clustalw2/). The crystal structure of dioscorin (PDB code: 4TWM) was uploaded as the secondary structure template.

YSP proteins were identified by LC-MS/MS and quantified using the emPAI label-free relative quantification method. There were 29 proteins identified from YSP, and 16 proteins showed emPAI values higher than 1.0 (Table 2). There were 15 dioscorin/tuber storage related proteins among the 16 high abundance proteins. Sequence alignment was conducted for these 15 proteins. As shown in Fig. 2, these proteins were all isoforms of dioscorin sharing high sequence similarity, which was similar to a previous study that identified 12 isoforms of dioscorin from a 2D gel of purified dioscorin^22^. Furthermore, acidic endochitinase (emPAI value: 12.74) was also detected in YSP, which provides defense against chitin-containing fungal pathogens^23^. Compared to dioscorin and its isoforms, however, the amount of acidic endochitinase was low. In addition, trace amounts of other proteins were also identified in YSP, including glyceraldehyde-3-phosphate dehydrogenase, ascorbate peroxidase and mannose-specific lectin.

**Table 2:** Proteins identified in YSP using the UniProt database.

Accession^a^	Score	Peptides (95%)	Mr	Protein name	Organism	emPAI
A7MAQ1	11148	29	30,749	**Dioscorin 4**	*Dioscorea japonica*	1798.6
A7MAQ2	10128	30	31,085	**Dioscorin 5**	*Dioscorea japonica*	1841.6
A7MAQ0	10016	28	30,723	**Dioscorin 3**	*Dioscorea japonica*	1623
Q75N34	9570	28	30,669	**Tuber storage protein**	*Dioscorea polystachya*	1222.6
A7MAQ3	9265	29	30,744	**Dioscorin 6**	*Dioscorea japonica*	1464.5
A7MAP9	7017	25	31,118	**Dioscorin 2**	*Dioscorea japonica*	816.34
A7MAP8	6958	26	31,136	**Dioscorin 1**	*Dioscorea japonica*	797.61
C7E3T9	6902	28	31,601	**Dioscorin**	*Dioscorea japonica*	399.4
C7E3T7	5673	24	31,333	**Dioscorin**	*Dioscorea japonica*	575.59
Q75N35	5672	25	31,232	**Tuber storage protein**	*Dioscorea polystachya*	392.11
Q39695	5522	18	31,222	**Storage protein**	*Dioscorea cayennensis*	128.06
Q9M4Z0	5030	20	31,134	**Dioscorin A**	*Dioscorea alata*	63.45
A0A1P8PPP9	4722	10	31,498	**Dio2**	*Dioscorea alata*	10.08
A0A1P8PPR6	3882	14	31,278	**Dio5**	*Dioscorea alata*	21.83
Q9M501	2905	12	31,560	**Dioscorin B**	*Dioscorea alata*	13.97
P80052	1886	11	28,859	Acidic endochitinase	*Dioscorea japonica*	12.74
Q852P9	567	4	32,310	Chitinase	*Dioscorea oppositifolia*	0.8
A0A1S5R1Z7	338	7	36,856	Glyceraldehyde-3-phosphate dehydrogenase	*Dioscorea oppositifolia*	0.83
A0A1J0MW81	192	4	40,154	Actin (Fragment)	*Dioscorea oppositifolia*	0.49
A0A2H4UZA8	94	2	27,354	Ascorbate peroxidase	*Dioscorea alata*	0.26
A0A2U9I6A9	27	1	40,043	Xanthine dehydrogenase (Fragment)	*Dioscorea x monandra*	0.08
A0A2I4PDC3	21	1	33,627	Maturase K (Fragment)	*Dioscorea fastigiata*	0.1
A0A0A0YSE7	19	1	62,275	3-hydroxy-3-methylglutaryl coenzyme A reductase	*Dioscorea composita*	0.05
Q5W956	17	1	16,108	Mannose specific lectin	*Dioscorea polystachya*	0.21
A0A024BK81	17	1	55,009	ATP synthase subunit alpha	*Dioscorea rotundata*	0.06
A0A024BKB8	16	1	56,284	Photosystem II CP47 reaction center protein	*Dioscorea rotundata*	0.06
A0A0A7E6J6	15	1	39,106	Rhomboid protein Dioop_PARL (Fragment)	*Dioscorea oppositifolia*	0.08
A0A1W6C8C7	14	1	222,527	Protein TIC 214	*Dioscorea villosa*	0.01
A0A097PQ43	14	1	36,321	BTB/POZ domain-containing protein (Fragment)	*Dioscorea oppositifolia*	0.09

^a^UniProt database accession numbers. Dioscorin and dioscorin isoforms are shown in bold font.

### Purification of YSP

**Figure 3:**
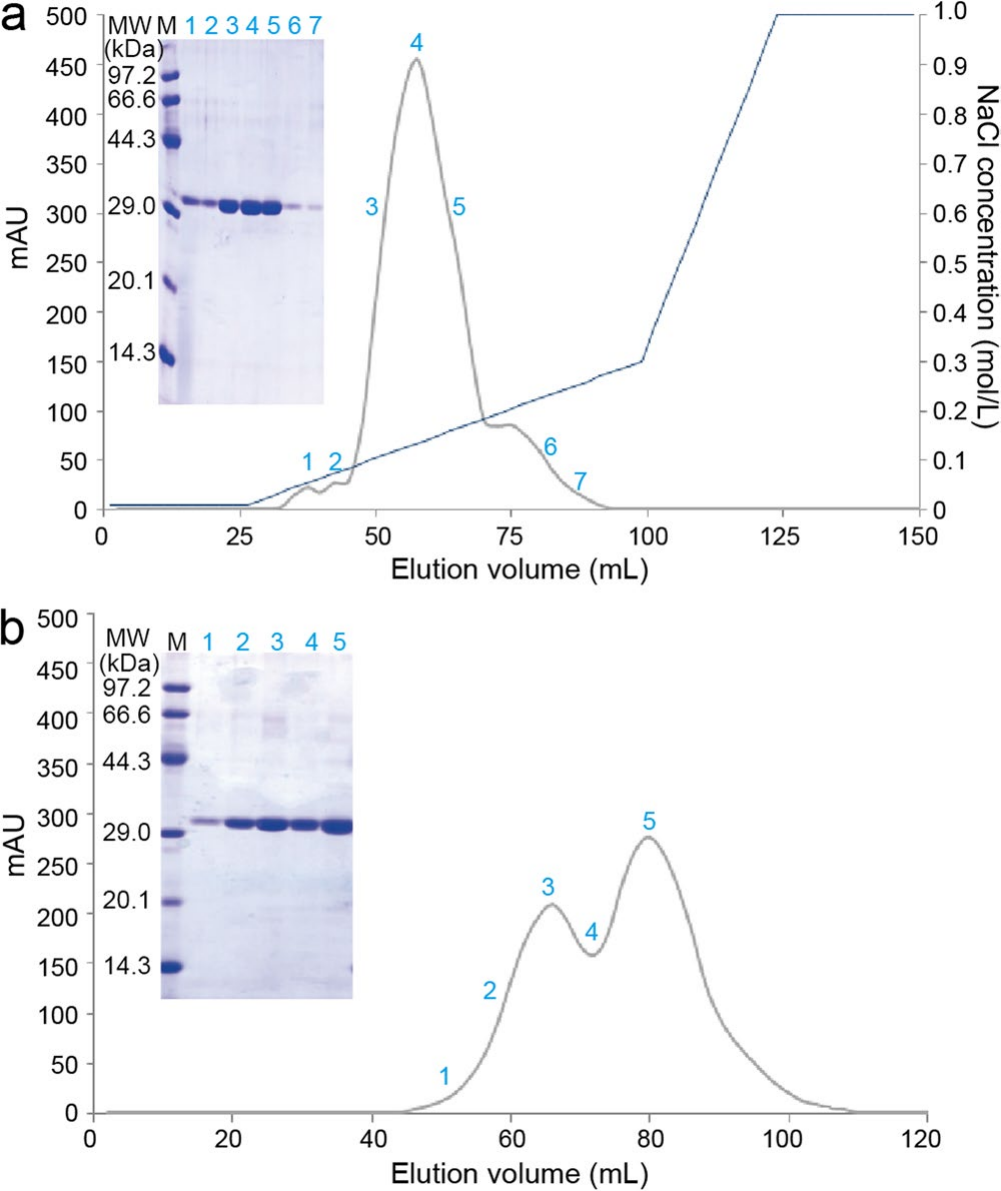
Anion-exchange chromatography (**a**) and size exclusion chromatography (**b**) of YSP using an AKTA FPLC system with Coomassie blue-stained SDS-PAGE gels. Left to right: Molecular weight (MW), marker (M) and Samples from chromatography.

The anion-exchange chromatography results of YSP are shown in Fig. 3a. A major peak was observed, and no other proteins besides dioscorin were detected on the SDS-PAGE gel, which was similar to our previous result^11^. The size exclusion chromatography result of YSP is shown in Fig. 3b. The elution profile shows that YSP was composed of two fractions, namely, a high molecular weight fraction and a low molecular weight fraction, which was similar to a previous report demonstrating that there are two classes of dioscorin (A and B) sharing 69.6% sequence identity^8^. Table 2 also indicates that there were mainly two molecular weights for dioscorin isoforms, namely, 30 and 31 kDa, which showed no difference on the SDS-PAGE gel (Fig. 3b; the original gels are shown in Supplementary Fig. 2). Thus, separation of the two fractions by size exclusion chromatography was not achieved.

Based on the separation results above, crude YSP without further purification, which is easily obtained by industrial scale recovery, was used for the following experiments.

### Amino acid composition of YSP

As shown in Table 3, the most abundant amino acids detected in YSP were Asp and Glu, accounting for 11.9% and 18.5% of the total amino acids, respectively. Collectively, the eight essential amino acids (Lys, Ile, Leu, Met, Thr, Phe, Val and Trp) of YSP made up approximately 33.7% of the total amino acids, which was slightly lower than that of sweet potato protein (40.7%)^24^. Some of these amino acids possess the following radical-scavenging activities due to their unique structure: the proton donation ability from the imidazole group of His; the hydrogen donation ability from the phenolic groups in Tyr and Phe; and the oxidization of Met to Met sulfoxide^25^. According to Table 3, the total content of the abovementioned four amino acid residues in YSP was 14.9% on a dry basis, which was higher than their combined contents in sweet potato protein (12.3%)^26^.

**Table 3:** Amino acid composition of YSP^a^.

Amino acid	Content	
	(mg/g dry weight)	% of YSP
Aspartic acid (Asp)	36.0 ± 0.2	11.86
Threonine (Thr)^b^	10.1 ± 0.1	3.33
Serine (Ser)	18.4 ± 1.1	6.06
Glutamic acid (Glu)	56.1 ± 0.8	18.48
Glycine (Gly)	11.1 ± 0.5	3.66
Alanine (Ala)	13.2 ± 0.3	4.35
Cystine (Cys)	12.0 ± 0.5	3.95
Valine (Val)^b^	15.4 ± 0.7	5.07
Methionine (Met)^b^	6.7 ± 0.8	2.21
Isoleucine (Ile)^b^	11.5 ± 0.3	3.79
Leucine (Leu)^b^	23.0 ± 1.2	7.58
Tyrosine (Tyr)	13.8 ± 0.7	4.55
Phenylalanine (Phe)^b^	18.2 ± 0.5	5.99
Lysine (Lys)^b^	15.8 ± 0.3	5.20
Tryptophan (Trp)^b^	1.6 ± 0.9	0.53
Histidine (His)	6.6 ± 0.2	2.17
Arginine (Arg)	32.6 ± 0.6	10.74
Proline (Pro)	1.5 ± 0.5	0.49
Total	303.6	100
% Essential amino acid	102.3	33.70

^a^The values reported represent the average of three determinations.

^b^Essential amino acids.

In addition, the content of some essential amino acids or pairs of amino acids in YSP preceded the standard of the ‘ideal protein’ described by the FAO/WHO^27^. Specifically, the contents of Ile, Met + Cys, Phe + Tyr and Val of YSP were 135%, 246%, 167% and 145%, respectively, of their requirements depicted by the WHO standard (Supplementary Table 2). However, the Lys, Thr and Tyr contents of YSP were lower than the FAO/WHO recommendation for children, accounting for 90%, 98% and 48% of the amino acid requirements, respectively.

### Free radical-scavenging activities of YSP

#### DPPH radical-scavenging activity

**Figure 4:**
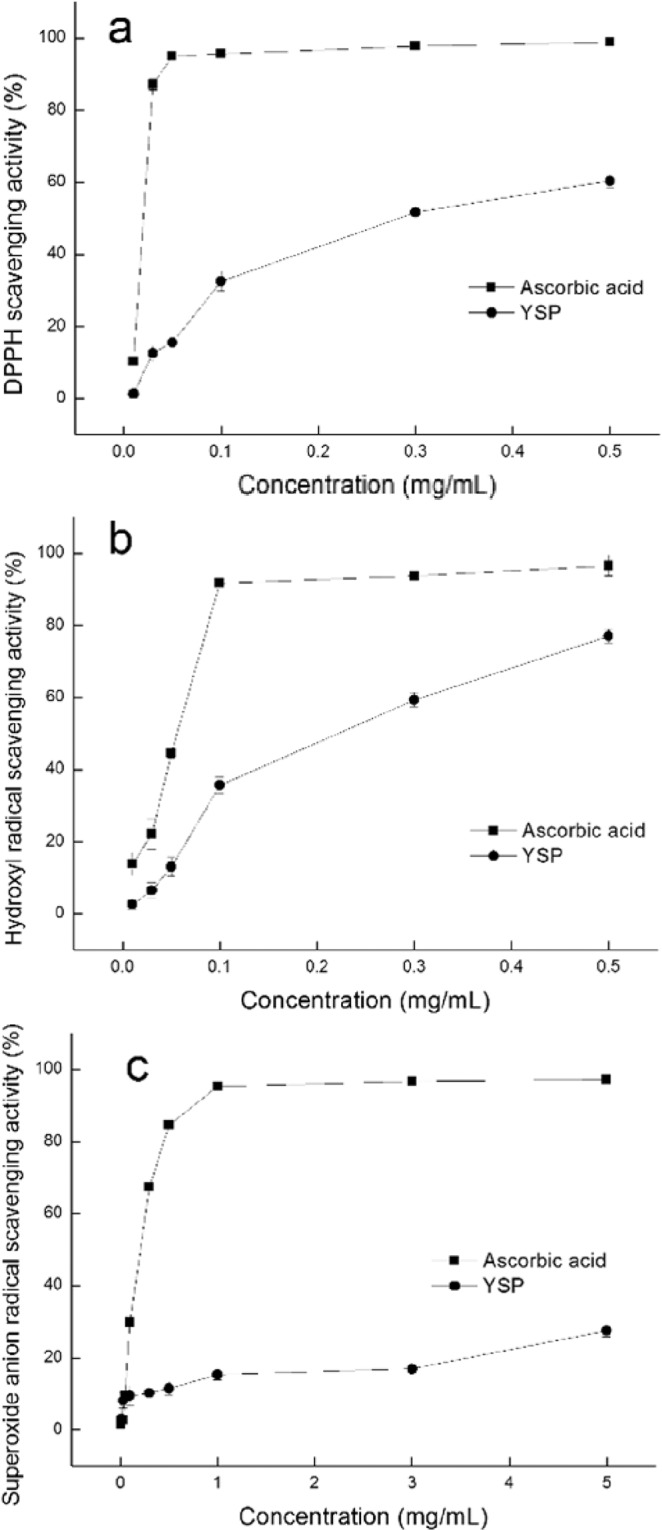
Scavenging activities of YSP and ascorbic acid to DPPH (**a**), hydroxyl radicals (**b**) and superoxide anion radicals (**c**) at different concentrations.

**Figure 5:**
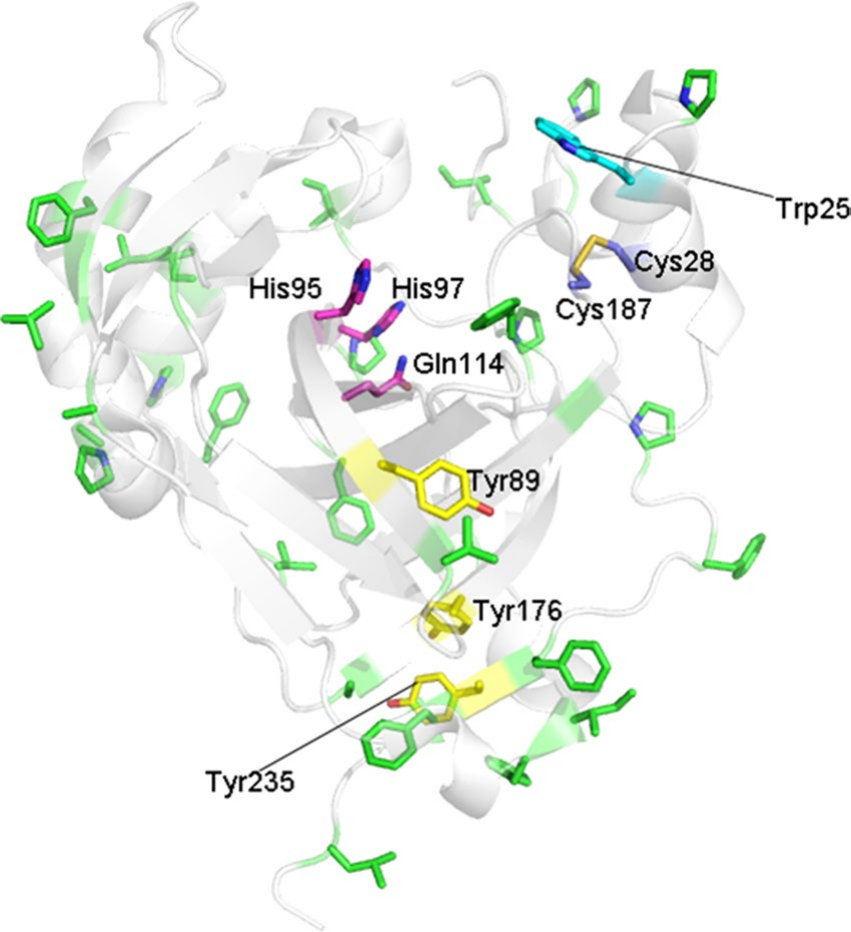
Amino acids and the disulfide bond are related to antioxidant activity. The amino acids are indicated by the following colors: cyan (Trp), yellow (Tyr), magenta (CA active center: His95, His97 and Gln114), blue (Cys28-Cys187 disulfide bond) and green (selected surface hydrophobic amino acids: Ala, Ile, Leu, Phe, Val, Pro and Gly).

The DPPH radical-scavenging activities of YSP and ascorbic acid were investigated (Fig. 4a). The results indicated that both YSP and ascorbic acid (positive control) showed evident scavenging activities on DPPH radicals in a concentration-dependent manner. The DPPH radical-scavenging activity of YSP increased from 1.31 to 60.4% when the protein concentration increased from 0.01 to 0.5 mg/mL. The DPPH scavenging activity of antioxidants was influenced by many factors. A previous report has suggested that the unpaired electrons of DPPH can react with protons donated by antioxidants to convert the DPPH radical into its nonradical form (DPPH-H)^28^. Hou *et al*. hypothesized that the thiol groups of yam storage protein (dioscorin) may reduce dehydroascorbate (DHA) to regenerate ascorbate to prevent oxidative damage to yam tubers^16^. Jheng *et al*. observed that there are no free thiol groups in dioscorin and that its DPPH radical-scavenging activity may be related to tryptophan, hydrophobic residues, or the hydroxyls of aromatic residues, such as tyrosine^18^. Structural studies have shown that dioscorin has a Cys28-Cys187 disulfide bond and that its DHA reductase activity is related to its carbonic anhydrase (CA) active center (Fig. 5)^29^. Thus, the DPPH-scavenging activities of YSP may be related to the aromatic amino acids (Trp and Tyr), the CA active center (His 95, His97 and Gln114) and some surface hydrophobic amino acids (Fig. 5).

#### Hydroxyl radical-scavenging activity

The scavenging activities of YSP and ascorbic acid (positive control) on hydroxyl radicals are shown in Fig. 4b. Both exhibited significant hydroxyl radical-scavenging activities that increased with increasing concentration (P < 0.05). The scavenging activity of YSP at 0.5 mg/mL was 77.1%, whereas the scavenging activity of ascorbic acid at 0.5 mg/mL was 96.6%. For YSP, the scavenging activity of hydroxyl radicals increased from 2.6% to 35.7% in the concentration range from 0.01 to 0.1 mg/mL. Tryptophan in patatin and sweet potato trypsin inhibitors have been reported to be responsible for hydroxyl radical-scavenging activities^19,30^. Figure 5 shows that Trp25 of dioscorin may be involved in the hydroxyl radical scavenging activity of YSP.

#### Superoxide anion radical-scavenging activity

The superoxide radical-scavenging activities of different concentrations of YSP and ascorbic acid (positive control) were tested (Fig. 4c). The scavenging activity of YSP at 5 mg/mL was 27.6%, whereas the scavenging activity of ascorbic acid at 5 mg/mL was 97.2%. For YSP, the scavenging activity increased from 3.2% to 15.4% in the concentration range from 0.01 to 1 mg/mL.

## Conclusion

YSP was successfully recovered from the wastewater of yam starch processing using the acid precipitation method. RSM was successfully performed on the extraction optimization of YSP. The results showed that YSP was mainly composed of dioscorin isoforms, which were rich in glutamic acid and aspartic acid. The eight essential amino acids made up approximately 33.7% of the YSP. Additionally, the present results showed that YSP had the function of scavenging free radicals. Further studies on the functional properties of YSP are required to expedite progress to make YSP an effective component in the nutraceutical and health food industries.

## Materials and Methods

### Materials

Yams (*Dioscorea opposita* Thunb.) were purchased from a local market (Shenyang, China)^31^. All reagents used in the present study were of reagent grade.

### Sample preparation

Starch production was conducted in the laboratory by imitating the starch factory process according to the method of Daiuto *et al*. with some modifications^32^. Yams were washed, peeled, sliced and crushed into a homogenate by adding different amounts of 1% sodium bisulfate solution (liquid-to-material ratio) at different pH values (extraction pH). The homogenate was stirred for a set period of time (extraction time) and filtered through a 0.25 mm sieve. After centrifugation at 5000 g for 30 min, the obtained supernatant was the wastewater that contained YSP.

The pH of the wastewater was adjusted to approximately 3.5 (the pH at which the largest amount of YSP is precipitated according to a preliminary study) using 2 mol/L HCl. After magnetically stirring for 1 h at room temperature, the slurry was centrifuged at 5000 g for 30 min. YSP was prepared by resolubilizing the pellet in distilled water with the addition of 1.0 mol/L NaOH to maintain a pH of 7, followed by ultrafiltration and lyophilization for other experiments. The concentrations of the proteins in the supernatants were measured according to Markwell *et al*.^33^. Protein extraction yield was calculated as follows: (the protein content of the supernatant)/(the protein content of the raw material) ×100.

### Parameter optimization for YSP recovery

First, the extraction pH, liquid-to-material ratio and extraction time were selected as the three variables to consider during the optimization (one-factor-at-a-time test) of the YSP extraction. Specifically, each variable was tested individually as follows: the pH was tested in a range from 7 to 11; the liquid-to-material ratio was tested in a range from 1:1 to 5:1 mL/g; and the extraction time was tested in a range from 0 to 80 min.

Second, the influences of the three variables, including (A) extraction pH, (B) liquid-to-material ratio and (C) extraction time, on the YSP yield (Y) were studied using RSM. Seventeen BBD runs were performed at all design points in a random order^34^.

### Proximate composition

The crude protein, total sugar, crude fiber, fat and ash contents of the YSP were determined according to the method of the AOAC (2005)^35^.

### SDS-PAGE

SDS-PAGE was performed according to the method of Shiu *et al*.^36^. The electrophoresis sample was prepared by dissolving YSP (1 mg/mL) in 2× sample buffer with β-mercaptoethanol (β-ME) and heating for 5 min in boiling water. Electrophoresis was performed on a 5% acrylamide stacking gel and 12% acrylamide resolving gel.

### Determination of YSP protein composition by LC-MS/MS

Protein bands were excised from the polyacrylamide gel and digested with 0.2 μg of trypsin (Promega V5280) in 20 μL of a 25 mM NH_4_HCO_3_ solution overnight at 37 °C. The peptides obtained were fractionated on an UltiMate 3000 HPLC system (Thermo Fisher Scientific, Sunnyvale, CA, USA) using a C_18_ column (Acclaim PepMap RSLC C_18_ 150 mm × 75 µm i.d., 2 μm). The 65 min gradients were performed at a flow rate of 300 nL/min starting from 5% to 35% solvent B (acetonitrile with 1% formic acid) over 40 min followed by a 5 min increase to 90% B, a 5 min maintenance period at 90% B and ultimately returning to 5% B. The peptides were subjected to positive ion nanoelectrospray ionization followed by MS/MS using a Thermo Scientific Q Exactive mass spectrometer. The original MS/MS data were converted to MGF formatted files using MM file conversion, and the obtained files were searched against the UniProt database using the MASCOT search engine. The following parameters were used for protein identification: carbamidomethyl (C) was the fixed modification; oxidation (M) was the potential variable modification with a maximum missed cleavage value (trypsin digestion) of 2, a peptide mass tolerance of 20 ppm and a fragment mass tolerance of 0.6 Da. The relative quantification of the identified proteins was accomplished using the exponentially modified protein abundance index (emPAI) label-free method^37^.

### Purification of YSP

#### Anion-exchange chromatography

YSP was first purified on a Resource Q anion-exchange column fitted to an AKTA FPLC system (GE Healthcare, Piscataway, NJ, USA). YSP solution (5 mg/mL; 5 mL) was injected into the system and eluted with a linear gradient of 0 to 0.3 mol/L NaCl in 0.02 mol/L Tris-HCl buffer (pH 8.0). The flow rate was 6 mL/min, and the main peak was collected and dialyzed for subsequent size exclusion chromatography.

#### Size-exclusion chromatography

YSP solution (10 mL) was injected into a Superdex 75 size exclusion column fitted to an AKTA FPLC system and eluted with 0.02 mol/L Tris-HCl buffer (pH 8.0). Each 3 mL fraction was collected at a 1 mL/min flow rate.

### Amino acid analysis

YSP (75 mg) was mixed with 10 mL of 6 mol/L HCl in a 20 mL ampoule. After sealing the ampoule, hydrolyzation of YSP was performed at 110 °C under vacuum for 24 h. The hydrolysate was evaporated to dryness at 60 °C under vacuum. Sodium citrate buffer (pH 2.2; 3–5 mL) was added to the dried sample to obtain a 50–250 nmol/mL amino acid concentration. The solution was then filtered and loaded on a Hitachi L-8800 amino acid analyzer (Hitachi Ltd., Tokyo, Japan)^24^.

### Determination of antioxidant activities

#### DPPH radical-scavenging assay

The DPPH radical scavenging activity was assayed using the method of Aluko and Monu^1^. In brief, 2 mL of diluted sample solution was mixed with 2 mL of DPPH solution (0.06 mg/mL). The solution was shaken vigorously and left to stand in the dark at room temperature for 20 min, and the absorbance was then measured at 517 nm. The scavenging activity was calculated by the following equation:

$${\rm{Scavenging}}\,{\rm{activity}}\,( \% )=[({{\rm{A}}}_{0}-{{\rm{A}}}_{1})/{{\rm{A}}}_{0}]\times 100$$

where A_0_ is the absorbance of the control group containing all reaction reagents without sample, and A_1_ is the absorbance of the reaction solution mixed with the test sample.

#### Hydroxyl radical-scavenging assay

Hydroxyl radical (•OH) scavenging activity was assayed as described by Sun *et al*.^38^. The hydroxyl radical was generated by mixing 1 mL of H_2_O_2_ solution (8.8 mmol/L) and 1 mL of FeSO_4_ solution (9 mmol/L). Then, 1 mL of salicylic acid solution (9 mmol/L) and 1 mL of the sample solution were added sequentially to the reaction system. The mixture was then shaken vigorously and incubated at 37 °C for 20 min, and the absorbance at 510 nm was measured. The scavenging activity was calculated by the following equation:

$${\rm{Scavenging}}\,{\rm{activity}}\,( \% )=[1\mbox{--}({{\rm{A}}}_{1}-{{\rm{A}}}_{2})/{{\rm{A}}}_{0}]\times 100$$

where A_0_ is the absorbance of the control group containing all reaction reagents without sample; A_1_ is the absorbance of reaction solution mixed with the test sample; and A_2_ is the absorbance of background value without H_2_O_2_.

#### Superoxide anion radical-scavenging assay

Superoxide anion radical scavenging activity was measured as described by Balavigneswaran *et al*.^39^. Briefly, 4.5 mL of 50 mmol/L Tris-HCl buffer (pH 8.2) and 1 mL of sample solutions at different concentrations were mixed and incubated at 25 °C for 20 min. The mixture was then shaken vigorously for 5 min after the addition of 0.2 mL of 60 mmol/L pyrogallic acid. Afterwards, 1 mL of 8 mol/L HCl was added to terminate the reaction. The absorbance was measured at 325 nm. The scavenging activity was calculated by the following equation:

$${\rm{Scavenging}}\,{\rm{activity}}\,( \% )=[1\mbox{--}({{\rm{A}}}_{1}-{{\rm{A}}}_{2})/{{\rm{A}}}_{0}]\times 100$$

where A_0_ is the absorbance of the control group containing all reaction reagents without sample; A_1_ is the absorbance of the reaction solution mixed with the test sample; and A_2_ is the absorbance of background value without pyrogallic acid.

### Statistical analysis

Data were analyzed by analysis of variance using the general linear model procedure of Version 8.0 SAS (SAS Institute Inc., NC, USA). Differences with probabilities of 0.05 or less were defined as significant. All experiments were performed in triplicate.

## Supplementary information

Supplementary Data.
