# Supplementary material for: Recovery of Yam Soluble Protein from Yam Starch Processing Wastewater
Source: Sci Rep. 2020 Mar 25;10:5384. doi: 10.1038/s41598-020-62372-6 (PMC7096408; doi:10.1038/s41598-020-62372-6)
Supplement: Supplementary file 1 — Supplementary Data. [file 41598_2020_62372_MOESM1_ESM.docx]

**Recovery of Yam Soluble Protein from Yam Starch Processing Wastewater**

Heng-Yue Xue^1,3^, Yue Zhao^1^, Zi-Heng Liu^1^, Xiao-Wen Wang^1^, Jun-Wei Zhang^1^, Xue Peng^1^, Masaru Tanokura^2,*^, You-Lin Xue^1,*^

^1^ College of Light Industry, Liaoning University, Shenyang 110036, P. R. China

^2^ Department of Applied Biological Chemistry, Graduate School of Agricultural and Life Sciences, The University of Tokyo, Tokyo 113-8657, Japan

^3^ Dalian Institute for Drug Control, Dalian 116021, P.R. China

**Supplementary data**

**Supplementary Table 1.** Data analysis results according to the Box-Behnken design

**Supplementary Table 2**. Essential amino acid composition of YSP compared to the WHO ‘ideal protein’

**Supplementary Fig. 1** Response surface for the effect of extraction pH (A), liquid-to-material ratio (B) and extraction time (C) on the yield of YSP.

**Supplementary Fig. 2** Full-length gels for Fig. 3.

**Supplementary Table 1.** Data analysis results according to the Box-Behnken design

| Number | A (pH of extraction  solution) | B (liquid-to-material ratio: mL/g) | C (Extraction time: min) | Yield (%) of YSP |
| --- | --- | --- | --- | --- |
| 1 | -1(8.5) | -1(2:1) | 0(30) | 50.234 |
| 2 | 0(9) | 0(3:1) | 0 | 52.293 |
| 3 | -1 | 1(4:1) | 0 | 55.543 |
| 4 | 1(9.5) | -1 | 0 | 40.506 |
| 5 | 0 | 1 | 1(40) | 50.076 |
| 6 | 1 | 0 | -1(20) | 46.529 |
| 7 | 0 | 0 | 0 | 51.975 |
| 8 | 0 | -1 | 1 | 45.625 |
| 9 | -1 | 0 | -1 | 57.740 |
| 10 | -1 | 0 | 1 | 56.743 |
| 11 | 0 | 0 | 0 | 53.083 |
| 12 | 1 | 0 | 1 | 43.910 |
| 13 | 0 | 1 | -1 | 51.026 |
| 14 | 0 | 0 | 0 | 52.767 |
| 15 | 0 | 0 | 0 | 52.609 |
| 16 | 1 | 1 | 0 | 42.695 |
| 17 | 0 | -1 | -1 | 48.825 |

**Supplementary Table 2**. Essential amino acid composition of YSP compared to the WHO ‘ideal protein’

| Amino acid | WHO ideal protein  (% of total protein) | YSP | |
| --- | --- | --- | --- |
|  |  | % of total  amino acid | % amino acid/  ideal protein × 100 |
| Ile | 2.8 | 3.79 | 135 |
| Leu | 6.6 | 7.58 | 115 |
| Lys | 5.8 | 5.20 | 90 |
| Met+Cys | 2.5 | 6.16 | 246 |
| Phe+Tyr | 6.3 | 10.54 | 167 |
| Thr | 3.4 | 3.33 | 98 |
| Trp | 1.1 | 0.53 | 48 |
| Val | 3.5 | 5.07 | 145 |


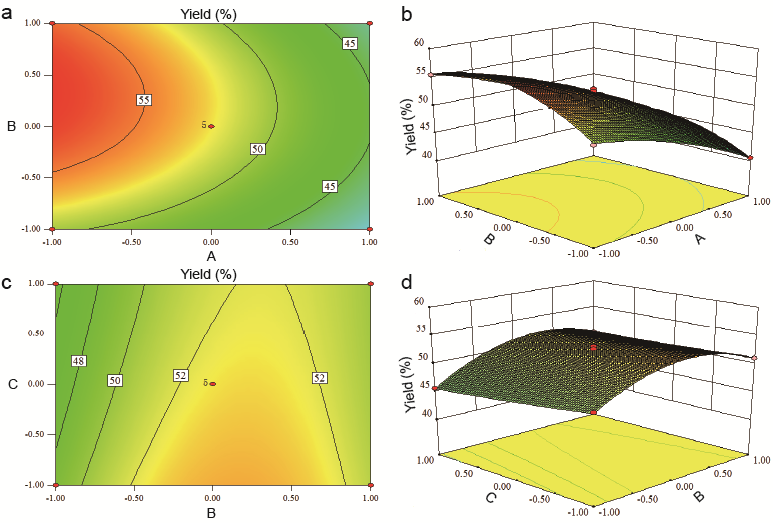


**Supplementary Fig. 1** Response surface for the effect of extraction pH (A), liquid-to-material ratio (B) and extraction time (C) on the yield of YSP.


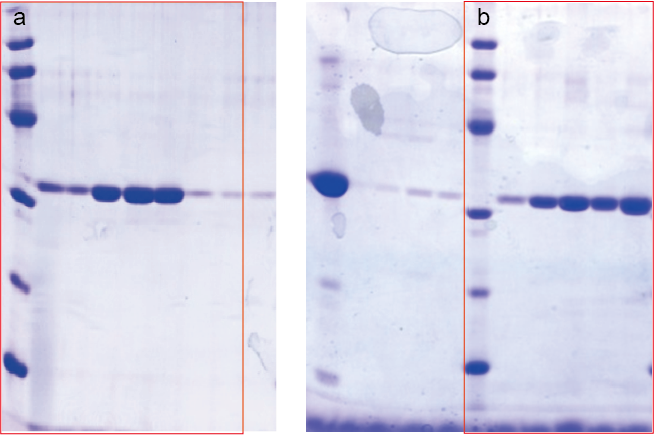


**Supplementary Fig. 2** Full-length gels for Fig. 3.
